# Behavioural and electrophysiological characterisation of experimentally induced osteoarthritis and neuropathy in C57Bl/6 mice

**DOI:** 10.1186/1744-8069-5-18

**Published:** 2009-04-20

**Authors:** Victoria L Harvey, Anthony H Dickenson

**Affiliations:** 1 Neuroscience, Physiology and Pharmacology, University College London, Gower St, London WC1E 6BT, UK

## Abstract

**Background:**

Osteoarthritis is a widespread condition affecting the elderly where ~70–90% of over 75 year olds are affected, representing one of the largest cost burdens to healthcare in the western world. The monosodium iodoacetate (MIA) osteoarthritis model has been well described in the rat especially in terms of the pathological progression of the disease and more recently pain behaviour. In this study, we characterise, for the first time, MIA induced osteoarthritis in mice and compare it with nerve-injured mice (partial sciatic nerve injury), using both behavioural and *in vivo *electrophysiological measurements. These approaches uniquely allow the threshold and suprathreshold measures to many modalities to be quantified and so form a basis for improving and expanding transgenic studies.

**Results:**

Significant mechanical hypersensitivity was observed in the ipsilateral hindpaw in MIA injected mice at all observed time points following infrapetellar MIA injection (p < 0.05). The mechanical hypersensitivity exhibited a partial biphasic temporal pattern, but thermal hypersensitivity was absent. Electrically-evoked dorsal horn neuronal responses in MIA injected mice were significantly elevated (p < 0.05) with respect to A- and C-fibre firing, input, pinch and noxious von Frey (26 and 60 g). No significant changes in A- or C-fibre thresholds were observed. Nerve-injured mice displayed significant behavioural thermal and mechanical hypersensitivity (p < 0.05) and evoked dorsal horn responses were significantly increased with respect to C-fibre firing, pinch and wind-up (p < 0.05).

**Conclusion:**

The MIA model of osteoarthritic pain in mice displays behavioural characteristics similar to those observed in rats. Changes in both behavioural measures and neuronal activity from the paw, suggest that central changes are involved in this pain state, although a role for peripheral drives is also likely. Moreover, the behavioural and neuronal measures in these two pain models showed overlapping alterations in terms of certain neuronal measures and mechanical sensitivity despite their very different pathologies and a loss of input in neuropathy, suggesting some commonalities in the central processing of different peripheral pain states. This murine model of osteoarthritis will allow the exploitation of knock out animals to better understand underlying mechanisms and identify novel molecular targets.

## Background

Chronic pain, caused by diseases such as arthritis or by nerve damage, affects millions of people worldwide. Osteoarthritis (OA) is a widespread condition affecting the elderly where ~70–90% of over 75 year olds are affected [1], and represents one of the largest cost burdens to healthcare in the western world accounting for 1–2.5% of their gross national product [2]. The majority of complaints concerning the disease are of chronic pain as well as a loss of joint function. Although the prevalence of neuropathic pain is difficult to ascertain due to its multifarious pathologies (e.g. postherpetic neuralgia, painful diabetic peripheral neuropathy, HIV polyneuropathy, etc.) it also presents a significant burden both to individuals and healthcare systems alike [3].

The majority of OA research has concentrated on the biochemical and anatomical pathology observed during the progression of the disease rather than studies involving the ensuing pain. Since there are no current therapies to slow the disease progression [4], analgesia is the first line treatment for OA. OA joint pain is described as a chronic and deep aching poorly localised pain, which is aggravated by physical activity and changes in the weather. Classical analgesics include paracetamol (acetaminophen), nonsteroidal anti-inflammatory drugs (NSAIDs), opioids and steroids. In the majority of patients, these treatments do not provide full pain relief and also display significant side effect profiles Although more is known with regards to the mechanisms underlying neuropathic pain [5,6], the knowledge is far from complete. Moreover, opioids, antidepressants and anticonvulsants (e.g. gabapentin) are used in the treatment of neuropathic pain but these therapies often display mixed efficacy in different patient groups. It is therefore important to try to elucidate the mechanisms responsible for the induction and maintenance of these pain states to help in the development of more effective analgesics for the treatment of OA and neuropathic pain. One such approach is to use transgenic mice to identify possible novel molecular targets. Success in these approaches requires robust and translational models.

The monosodium iodoacetate (MIA) OA model has been well described in the rat especially in terms of the pathological progression of the disease [7] and more recently pain behaviour [8,9]. Iodoacetate disrupts glycolysis by inhibition of glyceraldehyde-3-phosphate dehydrogenase, and subsequently causes chondrocyte death *in vitro *and *in vivo *[10]. This, therefore, represents an effective model of OA since it is thought that an imbalance occurs between the synthetic and degradative pathways within the articular cartilage resulting in abnormal cellular metabolism and that this represents a major causative factor of OA [11,12]. Since the structural integrity of cartilage relies on the normal functioning of chondrocytes, intra-articular injection with MIA produces cartilage degeneration and perturbations of the subchondral bone consistent with the clinical histopathology of OA [12-14]. As this degenerative model progresses, the subchondral bone becomes exposed generating joint impairment and associated pain [12] and mechanical hypersensitivity [8,9]. The pain-related behaviour in this model is thought to be characterised by an early acute inflammatory phase resulting from a fluid expansion of the synovial membrane followed by a persistent phase where the inflammation is largely resolved and is not thought to contribute to the pain pathogenesis [15].

The emphasis of this study concerns the pain associated with OA rather than the degenerative process *per se *since numerous papers have investigated the biochemical and pathological processes associated with this model [7,16]. Nevertheless, there are no studies that have compared both the behavioural and neuronal phenotypes of OA and neuropathy, a key to understanding the relations between pain mechanisms and symptoms in animals, especially the mouse where transgenic approaches can be used to reveal potential target mechanisms.

In this study we characterise, for the first time: MIA induced OA in C57Bl/6 mice using both behavioural and *in vivo *electrophysiological measurements; and compare the neuronal attributes of dorsal horn neuronal recordings in, partial sciatic nerve ligation (PSNL), a model of neuropathic pain. Whereas behaviour informs on threshold responses in awake animals, *in vivo *electrophysiology can quantify suprathreshold responses which can equate intensities that produce high levels of pain scores in patients.

## Results

### The MIA model of osteoarthritis

#### Animal development, general health, motor coordination and behaviour

No differences in body weight gain were observed between MIA-injected (22.7 ± 1.5 g, day 14) and saline-injected (21.4 ± 1.6 g, day 14) littermates at any time point throughout the study. The general health of the animals was good and no signs of obvious spontaneous pain behaviour, impaired motor function or distress were observed.

**Figure 1:**
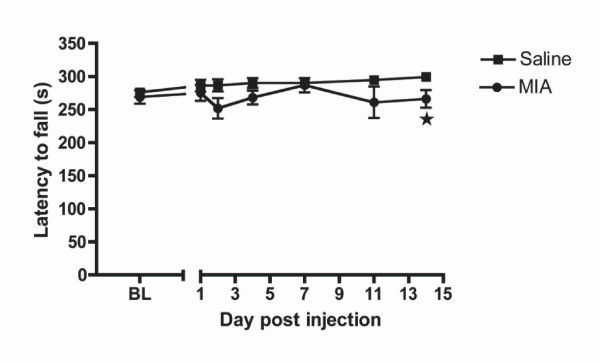
**Motor coordination of MIA-injected mice**. Latency to fall from the rotarod is expressed as mean ± S.E.M. Baseline (BL) measurements were measured 2–4 days before injection and expressed as a single mean value. * p < 0.05 compared with saline control animals (n = 10).

Motor coordination was assessed using the rotarod (Figure 1). Pre-injection (baseline) latencies to fall were similar in the saline (256 ± 6.9 s) and MIA-injected groups (269 ± 10.2 s; Figure 1). The mean latency to fall slightly increased over time suggesting that motor learning is present. A slight temporal biphasic trend, however, was observed for the MIA-injected mice when compared with the saline-injected mice; the mean latency to fall in the MIA-injected mice was significantly reduced at day 14 (266 ± 13 s) when compared with saline-injected mice (299 ± 0.5 s, p < 0.05, n = 10) (Figure 1). Although not quantified, no obvious differences in gait were observed at any time throughout the study.

**Figure 2:**
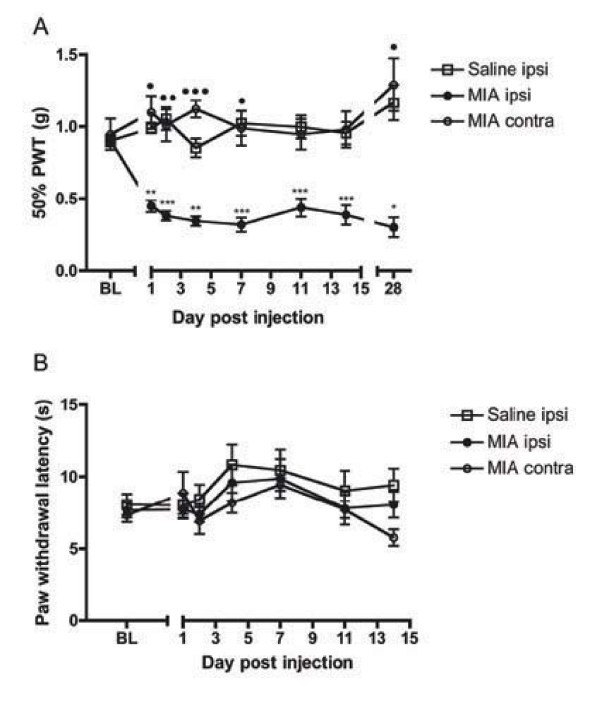
**Behavioural nociception in MIA-injected mice**. A. Development of mechanical hypersensitivity in MIA-injected mice. Graph depicts the changes in mechanical force required to elicit a paw withdrawal in response to a mechanical stimulus for the ipsilateral and contralateral hindpaws of MIA-injected mice (n = 18; day 28 n = 4) and the ipsilateral hindpaws of saline-injected mice (n = 16; day 28 n = 4). 50% paw withdrawal thresholds (PWT) are expressed as mean ± S.E.M. * denotes significant differences of ipsilateral MIA v ipsilateral saline; denotes significant differences of MIA ipsilateral v MIA contralateral. 3 symbols, p < 0.001; 2 symbols, p < 0.01; 1 symbol, p < 0.05. 2B. Lack of development of thermal hypersensitivity in MIA-injected mice. Graph depicts the time taken to elicit paw withdrawal in response to a noxious thermal stimulus for the ipsilateral and contralateral hindpaws of MIA-injected mice (n = 11) and the ipsilateral hindpaws of saline-injected mice (n = 11).

Behavioural mechanical hypersensitivity (mechanical allodynia) following MIA injection was assessed using the up-down method (Figure 2A). No significant changes from baseline were observed for contralateral MIA (data not shown) or ipsilateral saline responses (Figure 2A). Pre-injection (baseline) withdrawal responses were similar in the saline (0.90 ± 0.04 g) and MIA-injected groups (0.90 ± 0.03 g; Figure 2A). Significant mechanical hypersensitivity (i.e. a reduction in the force required to elicit a paw withdrawal) was observed in the ipsilateral hindpaw in MIA-injected animals at all observed time points following MIA injection (Figure 2A). MIA-injected mice displayed significantly decreased ipsilateral paw withdrawal thresholds when compared to ipsilateral saline (p < 0.001, n = 16) and contralateral MIA response thresholds (p < 0.01, n = 18) (Figure 2A). Mechanical hypersensitivity in the MIA-injected group displayed a slight temporal biphasic profile. The first phase of reduced withdrawal thresholds declined to a minimum at day 4 (0.34 ± 0.03 g) and 7 (0.32 ± 0.05 g), followed by a slight increase at day 11. Following this, withdrawal thresholds declined for a second time and persisted even when the study was extended to 28 days.

Behavioural thermal (heat) hypersensitivity (thermal hyperalgesia) following MIA injection was assessed using the Hargreaves' apparatus (Figure 2B). Pre-injection paw withdrawal latencies to a noxious stimulus were similar in both saline- (8.0 ± 0.8 s) and MIA-injected mice (7.7 ± 0.6 s) (Figure 2B). MIA-injected mice did not exhibit thermal hypersensitivity since the latency of hindpaw withdrawals to a noxious stimulus was not different from either pre-injection times nor latencies in saline injected animals (Figure 2B).

#### Spinal cord electrophysiology of dorsal horn neurones following MIA injection

Recordings were made from ipsilateral wide dynamic range dorsal horn neurones in MIA- (n = 12) and saline-injected mice (n = 13). No significant differences were observed for the mean depth of the neuronal recordings in the MIA- (517 ± 48 μm) and saline-injected neurones (508 ± 50 μm) thus allowing direct comparisons of the neuronal responses between the two groups. All of the cells had peripheral receptive fields on the plantar aspect of the hindpaw, where the most responsive areas were typically located in the toes. Electrophysiology experiments were performed between days 14–21 (post-injection), when the behavioural phenotype was persistent.

In the animals that received intra-articular injection of MIA, the responses to a train of 16 stimuli at 3 times C-fibre threshold, was found to have induced a significant facilitatory effect on A- and C-fibre-evoked responses and input (p < 0.05) but had no effect on A- or C-fibre thresholds (Table 1). Although not significant, a general facilitatory trend was observed for post-discharge and wind-up measures (Table 1). Wind-up, where dorsal horn neurones become hyperexcitable following repetitive C-fibre stimulation, was observed for all neurones in both groups. On comparing the rates of wind-up (i.e. Δ action potentials/stimulus no), Hill slopes for MIA-injected mice were significantly elevated compared to saline control animals (Table 1, p < 0.05).

**Table 1:** A comparison of dorsal horn neuronal responses evoked using electrical and natural stimuli in MIA and saline-injected mice

	MIA-injected	Saline-injected
A-fibre threshold (mA)	0.26 ± 0.05	0.25 ± 0.08
C-fibre threshold (mA)	1.07 ± 0.29	1.5 ± 0.25
A-fibre response (AP)	106 ± 15*	68 ± 10
C-fibre response (AP)	190 ± 35*	109 ± 13
Post discharge (AP)	215 ± 40	135 ± 20
Input (AP)	203 ± 34*	110 ± 22
Wind-up (AP)	257 ± 81	163 ± 37
Hill slope	24.3 ± 3.1*	17.2 ± 1.9
Brush (AP)	28 ± 7	36 ± 15
Pinch (AP)	145 ± 20*	86 ± 15
Acetone (AP)	14 ± 6	10 ± 5
1°C	19 ± 7	16 ± 5

Data are presented as mean ± S.E.M. for dorsal horn neurones recorded in MIA- (n = 12) and saline-injected mice (n = 13). AP represents the mean number of action potentials in response to either a peripheral electrical or 10 s natural stimulus. * p < 0.05.

Dorsal horn neuronal responses were also recorded in response to a wide range of natural stimuli. Neuronal responses to brush, non-noxious cooling (acetone), noxious cold (1°C water jet) were similar in the two groups (Table 1). Neuronal responses to noxious pinch, however, were facilitated in MIA-injected mice compared with saline controls (Table 1, p < 0.05).

**Figure 3:**
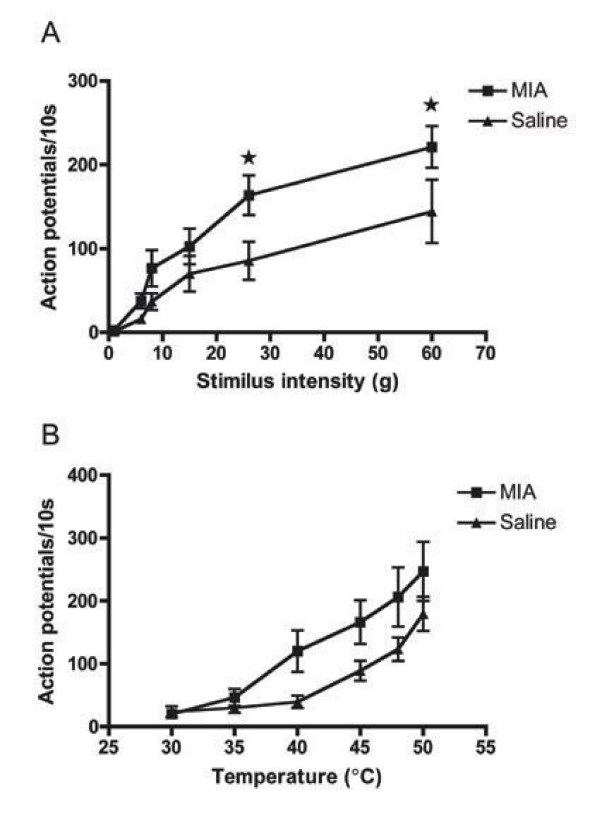
**Mechanical but not thermal coding of spinal neurones is facilitated in MIA-injected animals**. Stimuli were applied to the hindpaw for 10 s and responses are presented as mean ± S.E.M. (MIA, n = 12; saline, n = 13) A. Dorsal horn neurones displayed responses to mechanical stimuli in an intensity dependent manner. MIA-injected mice displayed exaggerated responses to noxious punctate mechanical stimuli * p < 0.05. B. Dorsal horn neurones displayed responses to thermal stimuli in an intensity dependent manner.

Spinal neuronal responses were then recorded in response to graded suprathreshold mechanical (1 – 60 g) and thermal stimuli (30 – 40°C water jet) (Figure 3A &3B). MIA-injected mice displayed enhanced evoked responses to punctate mechanical and thermal stimuli in an intensity-dependent manner. Although statistically significant effects were observed only for mechanical stimuli in the noxious range (von Frey 26 g and 60 g, p < 0.05), a trend for facilitation in response to thermal (heat) stimuli (Figure 3B) was also evident.

### The partial sciatic nerve ligation (PSNL) model of neuropathic pain

#### Animal nociceptive behaviour

**Figure 4:**
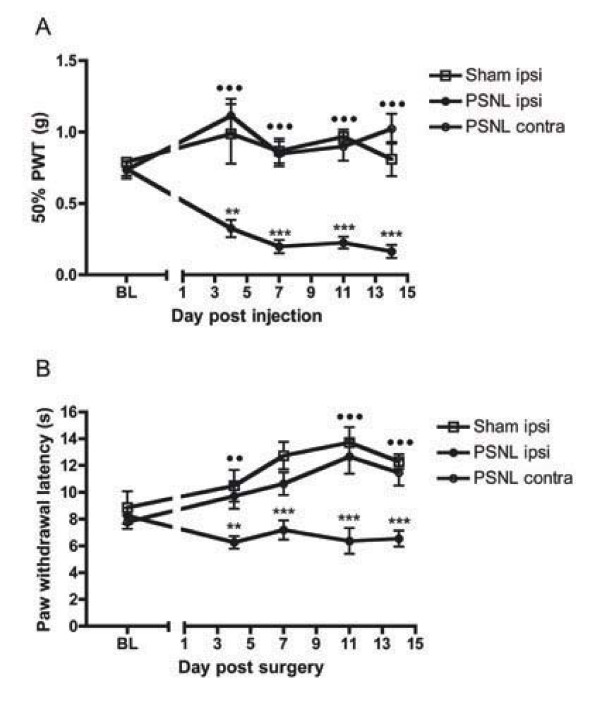
**Behavioural nociception in PSNL mice**. A. Development of mechanical hypersensitivity in PSNL mice. Graph depicts the changes in mechanical force required to elicit a paw withdrawal in response to a mechanical stimulus for the ipsilateral and contralateral hindpaws of PSNL mice (n = 11) and the ipsilateral hindpaws of sham mice (n = 11). 50% paw withdrawal thresholds (PWT) are expressed as mean ± S.E.M. B. Development of thermal hypersensitivity in PSNL mice. Graph depicts the time taken to elicit paw withdrawal in response to a noxious thermal stimulus for the ipsilateral and contralateral hindpaws of PSNL mice (n = 11) and the ipsilateral hindpaws of sham mice (n = 11). * denotes significant differences of ipsilateral PSNL v ipsilateral sham; denotes significant differences of PSNL ipsilateral v PSNL contralateral. 3 symbols, p < 0.001; 2 symbols, p < 0.01; 1 symbol, p < 0.05.

Mechanical hypersensitivity was assessed, as before, using the up-down paradigm (Figure 4A). No significant changes from baseline were observed for contralateral PSNL (data not shown) or ipsilateral sham responses (Figure 4A). Pre-surgery (baseline) withdrawal responses were similar in the sham (0.79 ± 0.03 g) and PSNL groups (0.74 ± 0.05 g). Significant mechanical hypersensitivity (i.e. behavioural mechanical allodynia) was observed in the ipsilateral hindpaw in PSNL animals at all observed time points following surgery (Figure 4A). Neuropathic mice displayed significantly decreased ipsilateral paw withdrawal thresholds to a punctate mechanical stimulus when compared to ipsilateral sham (p < 0.001, n = 11) and contralateral PSNL response thresholds (p < 0.001, n = 11) (Figure 4A), consistent with data reported from other groups.

Behavioural thermal hypersensitivity (thermal hyperalgesia) following PSNL surgery was assessed using the Hargreaves' apparatus (Figure 4B). Pre-surgery paw withdrawal latencies to a noxious stimulus were similar in both sham (8.9 ± 1.2 s) and PSNL mice (8.2 ± 0.6 s) (Figure 4B). No significant changes from baseline were observed for contralateral PSNL (data not shown) or ipsilateral sham responses (Figure 4B). Significant heat hypersensitivity was observed in the ipsilateral hindpaw in PSNL animals following surgery (Figure 4B). Neuropathic mice displayed significantly decreased ipsilateral paw withdrawal thresholds to a thermal stimulus when compared to ipsilateral sham (p < 0.001, n = 11) and contralateral PSNL response thresholds (p < 0.01, n = 11) (Figure 4A), consistent with other published data.

#### Spinal cord electrophysiology of dorsal horn neurones following PSNL surgery

Recordings were made from wide dynamic range ipsilateral dorsal horn neurones in PSNL (n = 13) and sham mice (n = 12). No significant differences were observed for the mean depth of the neuronal recordings in the PSNL (548 ± 23 μm) and sham mice neurones (543 ± 55 μm) thus allowing direct comparisons of the neuronal responses between the two groups. Depths and control responses of neurones in the PSNL animals were very similar to the comparable MIA groups. All of the cells had peripheral receptive fields on the plantar aspect of the hindpaw, where the most responsive areas were typically located in the toes. Electrophysiology experiments were performed between days 14–21 (post-surgery), when the behavioural phenotype was persistent.

In response to a train of 16 stimuli at 3 times C-fibre threshold, PSNL surgery was found to have a significant effect on C-fibre-evoked responses (p < 0.05), but no significant effects on any other electrical measure (Table 2). Similar to MIA, wind-up was observed in both neurones in PSNL and sham mice. Comparisons of Hill slopes revealed that PSNL surgery had a significant effect on the wind-up of dorsal horn neurones (p < 0.05; Table 2).

**Table 2:** A comparison of dorsal horn neuronal responses evoked using electrical and natural stimuli in PSNL and sham mice

	PSNL	Sham
A-fibre threshold (mA)	0.31 ± 0.07	0.27 ± 0.04
C-fibre threshold (mA)	0.77 ± 0.15	1.1 ± 0.21
A-fibre response (AP)	100 ± 11	89 ± 11
C-fibre response (AP)	166 ± 24*	101 ± 10
Post discharge (AP)	144 ± 30	99 ± 15
Input (AP)	211 ± 38	165 ± 29
Wind-up (AP)	104 ± 18	63 ± 12
Hill slope	24.8 ± 4.4*	15.1 ± 1.5
Brush (AP)	94 ± 21	67 ± 17
Pinch (AP)	237 ± 46**	80 ± 10
Acetone (AP)	18 ± 7	9 ± 3
1°C	77 ± 33	31 ± 15
Spontaneous (Hz)	3.4 ± 1.3	1.3 ± 0.8

Data are presented as mean ± S.E.M. for dorsal horn neurones recorded in PSNL (n = 13) and sham mice (n = 12). AP represents the mean number of action potentials in response to either a peripheral electrical or 10 s natural stimulus. * p < 0.05; ** p < 0.01.

Dorsal horn neuronal responses were also recorded in response to a wide range of natural stimuli. Neuronal responses to brush, non-noxious cooling (acetone), noxious cold (1°C water jet) were similar in the two groups (Table 2). Neuronal responses to noxious pinch, however, were facilitated in PSNL mice compared with sham controls (Table 1, p < 0.05).

**Figure 5:**
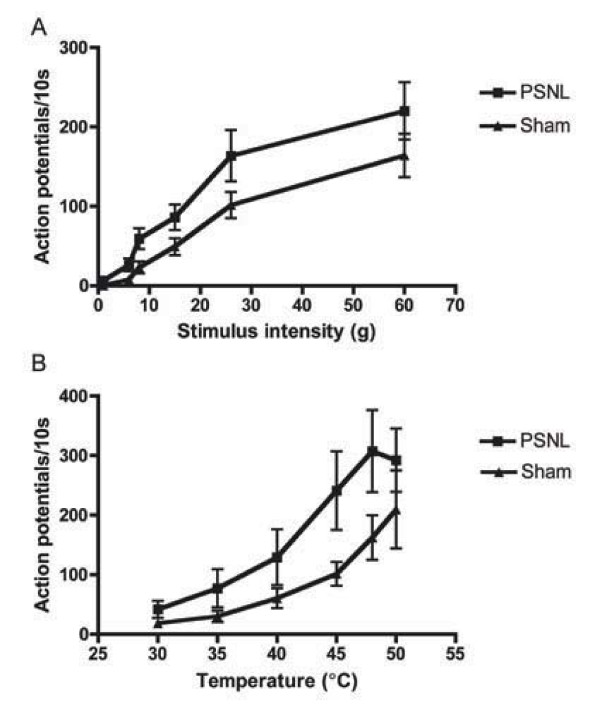
**Mechanical and thermal coding of spinal neurones is unaffected in PSNL mice**. Stimuli were applied to the hindpaw for 10 s and responses are presented as mean ± S.E.M. (PSNL, n = 13; sham, N = 12). A. Dorsal horn neurones displayed responses to mechanical stimuli in an intensity dependent manner. B. Dorsal horn neurones displayed responses to thermal stimuli in an intensity dependent manner.

Spinal neuronal responses were then recorded in response to graded suprathreshold mechanical (1 – 60 g) and thermal stimuli (30 – 40°C water jet) (Figure 5). Although neither mechanical nor thermal responses were significantly different from sham, a trend for facilitation in response to both mechanical (Figure 5A) and thermal (heat) stimuli (Figure 5B) was evident.

Increased stimulus-independent neuronal firing has been widely reported for neuropathic models in rats [17]. Although mean frequencies of stimulus-independent firing were increased, this was not significant (1.4 ± 2.4 Hz in sham and 3.4 ± 1.3 Hz in PSNL); 38% of neurones were spontaneously active in the PSNL group compared with 28% of those recorded from the sham group.

## Discussion

At present, the clinical treatment of OA is at best only partially effective and with the elderly population increasing, the prevalence of OA pain will rise requiring a greater need for clinically effective drugs to treat the pain associated with this disease. This highlights the need for effective models of OA pain in order to study OA pain pathogenesis and to identify novel therapeutic targets. The same holds for neuropathic pain. Here we report clear changes in behavioural and neuronal responses in the MIA model.

This study provides the first characterisation of MIA-induced OA in mice in terms of both pain behaviour and subsequent neuronal characterisation. The behavioural results, in this study, clearly demonstrate that a single infrapatellar injection of MIA into mice results in an early incidence of mechanical hypersensitivity on the hindpaw that is maintained throughout the testing period (28 days). The prevalence of behavioural mechanical hypersensitivity and absence of thermal hypersensitivity is consistent with the findings of other studies conducted in rats [9,16,18-20] and correlates well with clinical observations where, in some cases, patients report improvements in pain scores after application of heat or cooling packs. In rheumatoid arthritis in mice, induced using CFA, behavioural hypersensitivities are observed both for mechanical and thermal stimuli [21], thus highlighting the differences between these two disorders. Generally, OA has been regarded primarily as a noninflammatory arthropathy, however, local inflammation and synovitis are clinically reported and have been observed in animal models of OA. In this study, OA behaviour developed with a slight temporal biphasic profile. Although this early phase may represent the early synovial inflammation (synovitis), which contributes to the early development of OA [22]; it might be that the MIA itself is having a direct pro-inflammatory effect. Similar, but more marked, temporal patterns of OA behaviour have been observed following MIA injection in rats, where the first phase peaked at day 4 and was largely resolved by day 7, the second phase was initiated at day 14 and remained unresolved for the study duration [9,15,18,19]. In this study, therefore, the neuronal characterisation was performed from day 14, when the inflammation is thought to have been largely resolved and a more persistent pain state exists, since NSAIDs have previously only been found to be effective in the 3 days following MIA injection [8].

Although von Frey filaments were applied to the plantar aspect of the hind paw rather than the skin overlying the knee joint due to experimental hindrances; referred pain in the thigh, leg and foot of OA patients has been reported [23,24]. Cell bodies of afferents from the knee are thought to co-localise in DRGs with those of the hindpaw since retrograde labelling studies have shown that L3-L5 predominantly receive primary afferent input from the knee [25] and L3, L4 and L5 DRGs receive hind paw afferents [26]. This juxtaposition permits crosstalk such that pain transmitted by the afferents supplying the knee would engender pain in the hind paw. The behavioural studies of evoked pain in the hind paw support secondary hyperalgesia as this is characterised by mechanical hypersensitivity and often lacks heat hypersensitivity [27]. The spinal cord neuronal recordings bear out this premise in that measures of central hypersensitivity were observed.

Although cartilage itself is considered aneural, bone is densely innervated with Aδ and C fibres especially in regions of maximum load and high turnover such as those of the proximal and distal head of the femur [28]. During arthritis, pro-inflammatory agents such as bradykinin, substance P, and prostaglandins are released into the joint [29] which can lead to afferent fibre sensitisation and reductions in fibre thresholds (peripheral sensitisation). The establishment of OA in this study was accompanied by increases in electrically evoked A- and C-fibre responses with no changes in their activation thresholds. This might also be explained by the activation of silent nociceptors, which do not normally respond to stimuli but become active in response to tissue damage/inflammation [30]. These increases in A- and C-fibre firing may underlie OA patients' accounts of aching and throbbing pain interspersed with activity related sharp/stabbing pain [31]. The very clear increase in the non-potentiated 'input' response, indicative of increased peripheral drives, would result in an increased nociceptive drive onto dorsal horn neurons thus facilitating central mechanisms of hypersensitivity such as wind-up. The application of suprathreshold stimuli, during the electrophysiological characterisation, evoked enhanced responses to noxious mechanical stimuli. This electrophysiology is similar to a study on CFA-induced arthritis in the rat, where facilitated mechanical responses in the noxious range and increases in the proportion of neurones responding to mechanical stimulation were observed in neurones receiving input for the joints [32]. Thus the increased input, wind-up, A- and C-fibre firing, response to pinch and mechanical hypersensitivity observed both in the behaviour and electrophysiology imply a role for central sensitisation in the MIA model of OA pain.

Although, peripheral neuropathic trauma may induce the release of inflammatory mediators this is only likely in the early stage of neuropathic pain. Likewise, osteoarthritic degeneration could lead to nerve compression, a common cause of neuropathic pain but again, this is not likely to be a major mechanism. It is likely that OA initiates an inflammatory state as cartilage degrades and then moves to a nociceptive pain in the later stages with direct physical stress on bones providing the peripheral drive. Neuropathic pain, by contrast, involves disordered and altered ion channel and other functions in the damaged and spared peripheral nerves and is so restricted to the nerve territory and involves both loss and gain of function. Both the behavioural and neuronal measures in these models show overlapping alterations produced by the very different pathologies. This suggests some commonalities in the central processing of different peripheral pain states but with marked upregulations in the neuropathy model, possibly compensating for the afferent fibre loss [33]. Mechanical hypersensitivity was marked in both models but greater in PSNL, perhaps relating to the segmental changes in the nerve injured zone, where testing was done. In the MIA model the enhanced mechanical responses are likely to represent referred pain since the hindpaws were tested after knee MIA. Both groups showed larger mechanical evoked responses than their controls, significant in the MIA group. Input was enhanced in the MIA group but unchanged after nerve injury, perhaps reflecting the loss of input caused by the neuropathy yet enhanced primary drives onto deep dorsal horn neurones after MIA. The trend towards an increased thermal responses of neurones was not reflected in the behaviour and this is likely a result of the suprathreshold nature of the neuronal responses – the responses of the neurones to lower temperatures, akin to the behaviour were not altered.

Clinical observations have also suggested central changes in nociceptive information processing in patients with OA. Support for this comes from a study conducted in patients with bilateral symptomatic OA of the knee, where a single intra-articular injection of bupivacaine (local anaesthetic) into the most painful knee caused a reduction in pain scores for both the ipsilateral and contralateral knee [34]. In this study, it seems likely that there is an important central involvement in OA pain as typical indicators for peripheral sensitisation, namely decreased activation thresholds for A- and C-fibres (where stimuli would bypass sensitized peripheral terminals), were not observed in the neuronal characterisation, although an increase in input was observed that could reflect augmented excitability. A role for peripheral sensitisation or altered peripheral drives cannot, therefore, be ruled out and may be more important during OA pain induction rather than its maintenance.

At early stages of this model in rats, NSAIDs display good efficacy but become ineffective at later time points [8,15,18], and pain behaviour can only be attenuated using morphine [18] and Gabapentin [16]. Furthermore, repeat dosing studies, a regimen more consistent with the clinical setting, found that both gabapentin, used for neuropathic pain in patients, and morphine displayed efficacy in this model [9]. This pharmacology suggests that the early stages of the model/disease reflect acute inflammatory pain, but as the disease progresses the inflammation is largely resolved and starts to share similarities with those of neuropathic pain. Furthermore, in one study, a significant increase in the levels of ATF-3 (a marker for neuropathy) in L5 dorsal root ganglia was found at days 8 and 14 following MIA injection in rats, however, no differences were found at later time points up to days 35 [16].

We demonstrate increases in C-fibre evoked responses and wind-up of WDR neurones in PSNL mice. Along with the slight increase in the proportion of neurones displaying spontaneous activity (and at a slightly higher rate), these changes are consistent with those expected for central hyperexcitability. Similarly, a trend was observed for thermally and mechanically evoked responses, although this did not achieve significance. This is consistent with electrophysiological studies performed in spinal nerve ligated rats, where L5 and L6 dorsal roots are tightly ligated reducing spinal afferent input to the hindpaw to 30–40% [35], but comparable responses are observed in the sham groups to peripherally applied stimuli [36-38]. This suggests, in concordance with the rat, that these remaining hyperexcitable neurones can compensate for the sensory loss occurring in these pain states. Following nerve injury, α_2_δ subunits of voltage gated calcium channels are slowly upregulated in the central terminals of peripheral nerves leading to spinal hyperexcitability [39], spinal neurones receive greater descending facilitatory influences [36] and receptive fields expand [40]. The former changes are permissive for the actions of gabapentin and pregabalin [41] and may therefore also reflect both the spinal hyperexcitability observed in OA in this study and the efficacy of gabapentin observed in rat studies of OA [9]. Studies of this nature in the MIA model will allow further comparative mechanistic studies across different pain aetiologies.

## Conclusion

Increasing evidence suggests that OA is not a single disease entity, rather that it is a syndrome arising from a group of disorders with similar pathologies. It is, therefore, naïve to believe that OA pain can be simply classified and thus it can be described both by inflammatory and neuropathic pain characteristics. Furthermore, the degenerative changes associated with OA might lead to compression of the nerves and may result in peripheral neuropathy. However, OA pain differs to that of neuropathic pain as in the majority of OA patients, hyperalgesia is abrogated once the affected joint has been replaced [27], whereas neuropathic pain is thought to outlast its primary cause. The central hypersensitivity in OA is likely to be at least part mediated by chronic nociceptive pain which is thought reversible following the removal of the peripheral nociceptive drive.

From this study and others, it would seem that OA pain represents a distinct pain entity characterised by both early inflammatory and late persistent pain. Although neuropathic pain and OA pain are likely to be mediated by divergent peripheral mechanisms, this study suggests that they share some common central mechanisms. It would be interesting, therefore, to investigate the role of descending facilitation and α_2_δ subunits in OA pain, two key modulatory mechanisms of neuropathic pain [33,38,42]. These results suggest that central sensitisation underlies the behavioural mechanical hypersensitivity observed in this model of OA. This murine MIA model of OA-induced pain, therefore, represents a rapid and reliable tool for identifying novel therapeutic targets especially when there are no available pharmacological ligands.

## Methods

All procedures were performed according to current UK home office legislature (Animals Scientific procedures Act, 1986). Adult (8–12 weeks; 20–30 g) C57Bl/6 mice of mixed gender were used for these studies (Harlan, UK). Animals were housed in groups of 3–4, with 12 h light/dark cycle and allowed food and water *ad libitum *except during procedural testing. Surgical procedures were performed under general anaesthesia with halothane (1–2%) delivered in O_2_.

### Monosodium iodoacetate (MIA) injection

OA was induced, in briefly anaesthetised mice, by a single intra-articular injection of MIA (Sigma, UK) into the knee. Knee joints were shaved and flexed at a 90° angle; 5 μl of 5 mg/ml MIA in sterile saline (0.9%) was injected through the infra-patellar ligament into the joint space of the left (ipsilateral) knee using a 30-gauge 0.5" needle. This concentration of MIA has been found previously, in mice, to precipitate histological changes in the cartilage, consistent with those of osteoarthritis [43]. Control mice received an intra-articular injection of vehicle (5 μl sterile saline, 0.9%).

### Partial Sciatic Nerve Ligation (PSNL)

Nerve injury was induced in deeply anaesthetised mice using a method based on that previously described by [44,45]. The left (ipsilateral) sciatic nerve was exposed above its trifurcation and one third to one half of the nerve was tightly ligated using 7-0 non-absorbable silk suture (Mersilk, Ethicon). The wound was closed in layers using 4-0 suture (Mersilk, Ethicon), and animals were allowed to recover. In sham mice, the sciatic nerve was exposed, but not ligated and was closed as before.

### Nociceptive behaviours

All behavioural testing was preceded by two baseline measurements taken 2–4 days prior to injection/surgery. A single observer was used for the study, and was blind to the treatment given to each animal.

### Behavioural tactile hypersensitivity (von Frey hairs)

Animals were placed in a Perspex chamber with a wire mesh floor, and allowed to acclimatise for at least 2 hours prior to testing. Tactile hypersensitivity was tested by touching the plantar surface of the hindpaw with von Frey filaments using the "up-down method" [46], starting with 0.6 g then ranging from 0.07 g to 1.4 g. Positive withdrawals were counted as biting, licking and withdrawal during or immediately following the 3 s stimulus. The strength of the von Frey filament was increased or decreased following a negative or positive response respectively. This up-down procedure was applied 4 times following the first change in response and stimuli were not re-applied within a 5 minute period. Data are presented as 50% paw withdrawal threshold (PWT) for each group ± SEM.

### Heat hypersensitivity (Hargreaves'test)

Mice were assessed for thermal hypersensitivity as described by [47]. Mice were placed in translucent chambers and were allowed to acclimatise for at least 1 hour. Noxious heat sensitivity was measured using a radiant heat device directed at the plantar aspect of the paw. Paw withdrawal latencies, accurate to the 0.1 s were noted, where a maximal cut off time of 20 s was used to minimise paw damage. The stimulus intensity was adjusted to ~8 s withdrawal latency for naive animals. Measurements were taken 3–5 times with at least 5 minutes between tests. Data are presented as mean withdrawal latency for each group ± SEM.

### Rotarod

Animals (n = 10 per group) were placed on the rotarod at a speed of 4–40 rpm for a maximum of 300 s. During baseline tests two training trials were given followed by two recorded trials. Two sets of baseline values were recorded and their mean was taken. Animals were excluded from further testing if they had not achieved a trial of > 250 s in any of their 8 baseline trials. After surgery, three trials were performed and the mean latency was recorded. Animals were allowed to rest for at least 15 min between testing. Data are represented as mean latency to fall for each group ± SEM, where the latency to fall for mice freely rotating on the drum was recorded following two successive rotations.

### Spinal cord electrophysiology

*In vivo *electrophysiology was performed at days 14–21 following MIA/sham injection or surgery using parylene coated tungsten electrodes (A-M Systems, USA). Animals were anaesthetised using urethane (240 mg/Kg), and a laminectomy was performed exposing L3-L5 of the spinal cord. Once a single wide dynamic range neurone had been isolated in the ipsilateral dorsal horn receiving inputs from the hindpaw, A- and C-fibre thresholds were measured and noted. A train of 16 electrical stimuli (2 ms wide pulses, 0.5 Hz), delivered transcutaneously by means of pins inserted in to the hindpaw, was applied at 3 times the activation threshold for C-fibre responses. A post stimulus time histogram was constructed and fibre responses were separated according to latency: A (0–50 ms) C (50–250 ms). Responses occurring after the C fibre latency band were characterised as post discharge (250–800 ms). Input (non-potentiated response) was calculated as the number of action potentials in response to the first stimulus × total number of stimuli (16). Wind up, a measure of the neuronal activity in response to a succession of stimuli and hence synaptic strength, was calculated as the total number of action potentials evoked at the end of the train – the input. A wide range of natural stimuli including brush, von Frey filaments and heat were applied to the hindpaw for a period of 10 seconds. Data were captured and analysed using a CED 1401 interface coupled to a Pentium computer running Spike 2 software (Cambridge Electronic Design).

### Data analyses

Data are expressed as mean ± SEM, where n represents the number of individual experiments performed. Raw data were analysed using two-way repeated measure ANOVAs, Kruskal-Wallis, Mann-Whitney and unpaired student T tests as appropriate where p < 0.05 was considered significant.

## Competing interests

The authors declare that they have no competing interests.

## Authors' contributions

VLH and AHD conceived, designed and performed the experiments, analysed and wrote the manuscript.
